# Evaluation of Myoelectric Activity of Paraspinal Muscles in Adolescents with Idiopathic Scoliosis during Habitual Standing and Sitting

**DOI:** 10.1155/2015/958450

**Published:** 2015-10-25

**Authors:** Garcia Kwok, Joanne Yip, Mei-Chun Cheung, Kit-Lun Yick

**Affiliations:** ^1^Institute of Textiles and Clothing, The Hong Kong Polytechnic University, Hung Hom, Kowloon, Hong Kong; ^2^Department of Social Work, The Chinese University of Hong Kong, Shatin, New Territories, Hong Kong

## Abstract

There is a number of research work in the literature that have applied sEMG biofeedback as an instrument for muscle rehabilitation. Therefore, sEMG is a good tool for this research work and is used to record the myoelectric activity in the paraspinal muscles of those with AIS during habitual standing and sitting. After the sEMG evaluation, the root-mean-square (RMS) sEMG values of the paraspinal muscles in the habitual postures reflect the spinal curvature situation of the PUMC Type Ia and IIc subjects. Both groups have a stronger average RMS sEMG value on the convex side of the affected muscle regions. Correction to posture as instructed by the physiotherapist has helped the subjects to achieve a more balanced RMS sEMG ratio in the trapezius and latissimus dorsi regions; the erector spinae in the thoracic region and/or erector spinae in the lumbar region. It is, therefore, considered that with regular practice of the suggested positions, those with AIS can use motor learning to achieve a more balanced posture. Consequently, the findings can be used in less intrusive early orthotic intervention and provision of care to those with AIS.

## 1. Introduction

Adolescent idiopathic scoliosis (AIS) is a multifactorial, three-dimensional deformity of the spine and trunk. It can appear and sometimes progress during any of the rapid periods of growth in children [1]. Noninvasive brace therapy is usually recommended for spinal curvatures between 21 and 40 degrees while surgery is suggested for curvatures over 41 degrees [2, 3]. Conventional orthoses are used to apply passive forces onto the human body to support the trunk alignment and control the deformity of the spine. However, the use of an external support is affected by factors such as poor appearance, bulkiness, and physical constraint that could lead to low acceptance and compliance [4, 5]. Regardless of current clinical practices, treatment is nothing more than just observation if the curve is less than 20 degrees, even if the child is at high risk of progressive spinal deformity during puberty, which is between the ages of 10–16 [6, 7]. The reason is that the prediction of curve progression is not available for untreated AIS patients (a Risser stage less than 1 is 22% and larger than 1 is only 2–4%) [8] and alternative treatment options are very limited. However, with biofeedback as an area that is eliciting growing interest in the medical and psychology fields, and its proven effectiveness for a number of physical, psychological, and psychophysical problems [9–11], it is possible that biofeedback can be one of the new techniques that will provide an alternative type of noninvasive treatment for AIS patients.

Biofeedback is a nonmedical process that involves the measuring of specific and quantifiable bodily functions of a subject, such as the brain wave activity, blood pressure, heart rate, skin temperature, sweat gland activity, and muscle tension, thus conveying the information to the patient in real-time. The basic aim of biofeedback therapy is to support a patient in realizing his/her self-ability to control specific psychophysiological processes [12]. The literature has consistently indicated that surface electromyography (sEMG) biofeedback is effective for muscle rehabilitation. A review of the sEMG studies on upper extremity dysfunctions in the physically disabled [13] concluded that sEMG is a valuable method for increasing upper extremity muscle activity and most effective when used in conjunction with physiotherapy. In a meta-analysis of sEMG biofeedback studies applied to hemiplegic stroke subjects, Schleenbaker and Mainous III concluded that the use of sEMG improves functional outcomes in both the upper and the lower extremities and that sEMG should be included in therapeutic regimes [14]. Therefore, a thorough evaluation of the myoelectric activity in the muscles of those with AIS would be extremely important for the formulation of a database towards sEMG biofeedback training.

Different studies have also been done on the paraspinal muscle activity of adolescents with idiopathic scoliosis by using sEMG. Avikainen et al. recorded the force-time and the EMG-time curves of the paraspinal muscles during maximal isometric trunk extensions. The isometric force-time curves as well as the maximal integrated EMG activity that were recorded from both sides of the thoracic and lumbar spines did not show any significant differences between the normal and scoliotic groups [15]. Chwała et al. also conducted an EMG assessment of the paraspinal muscles during static exercise in those with AIS. They found that, during symmetric and asymmetric exercises, the muscle tension patterns significantly differ for both the normal and scoliotic groups in comparison with the examination at rest, which in most cases generated positive corrective patterns [16]. Farahpour et al. investigated the EMG activity of the erector spinae and external oblique muscles during lateral bending and axial rotation between AIS patients and a healthy control group. Their findings showed that asymmetric muscle activity is not obvious in all of the tested postures; therefore, asymmetric muscle activity is not a necessary characteristic of AIS patients [17]. Odermatt et al. examined the EMG signals of the trunk muscles in braced and unbraced conditions under four specific trunk exercises. The results showed that the tested muscle area under the braced condition has a significant increase of 43% in EMG activity for three out of four exercises [18]. The mentioned studies aimed to test the paraspinal muscles under specific motions or exercises. Although the motions and exercises tested in these studies can reflect how the muscle activity of AIS patients is different from that of healthy subjects, two daily postures that are commonly performed, that is, standing and sitting, have not been included. Nault et al. investigated the difference in the standing stability between 71 able-bodied girls and subjects with AIS. The scoliotic group had a larger number of correlations between standing stability and posture parameters than the nonscoliotic group which indicates the standing imbalance of the scoliotic group [19]. The study showed that scoliotic patients have issues in maintaining standing stability. However, it was compared by using center of pressure displacements. The performance of the paraspinal muscles was not included in the study. Our approach is different. We have conducted a thorough evaluation of the myoelectric activity of those with AIS during habitual standing and sitting by using sEMG. Also, we have studied the changes in the myoelectric activity of the paraspinal muscles after a position has been suggested by a physiotherapist so as to investigate the muscle activity of scoliosis subjects during their most performed motions, that is, standing and sitting, and the difference between the habitual and suggested positions. The findings can thus be used to provide a less intrusive type of early intervention and serve as a means of care for adolescents with mild idiopathic scoliosis (i.e., Cobb's angle less than 20 degrees) and therefore reduce the possible need to prescribe brace wear treatment due to its associated psychological issues and negative impacts on adolescents.

## 2. Methods

**Figure 1:**
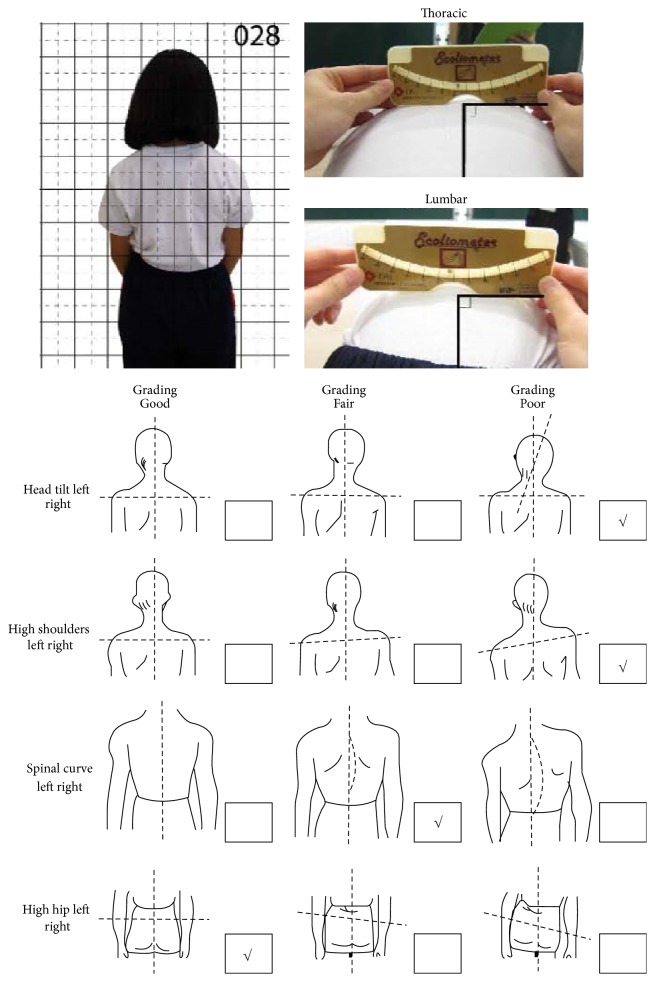
Adam's forward bending test and posture record form.

A screening program was carried out in Hong Kong during 2014 with 2 schools and the target population was 10–13-year-old females. During the examination process, the subjects were invited to perform Adam's forward bending test and an OSI scoliometer was employed to measure the rib hump which is directly related to spinal rotation and rib deviations. The angle of trunk rotation (ATR) in the spine of the subjects was measured while lying prone in order to preliminarily assess their spinal conditions (as shown in Figure 1). The participants were assigned to the normal subject group (N group) if they had an ATR 0–2° without any posture problems. They were assigned to the group with signs of scoliosis (P group) if they had an ATR ≥ 3°. This is because an ATR > 3° might be an early sign of scoliosis and the concerned individuals were recommended to undergo checkups more frequently [20]. In total, out of the 185 participants who were screened, 26 were found to have an ATR ≥ 3° (14.1%).

Participants from the P group accepted the invitation to take lateral 3D images through ultrasound by using the Scolioscan [21, 22]. Clinicians use the Scolioscan to measure the spine deformity angle and rotation through manually assigned markers on 3D images. This method is considered to be potentially compatible with the traditional Cobb's angle measurement which uses X-rays and yet the subjects do not face a radiation hazard [23]. After the evaluation, a total of 21 participants with a curve angle of 6 to 20 degrees, without any previous surgical or orthotic treatment for AIS, were recruited for the study. The study was approved by the Human Ethics Committee of the Hong Kong Polytechnic University. All of the subjects signed an informed consent form along with their parents, and both were informed about the purpose of the study.

Curve type in this study is defined on the basis of the guidelines from the Peking Union Medical College (PUMC) classification system [24]. The subjects (*N* = 21) were divided into 3 groups: PUMC type Ia, a single thoracic curve with apex between the T2 and T11-T12 disc; PUMC type Ib, a single thoracolumbar curve with apex at T12, T12-L1 disc, and L1; and PUMC type IIc (double curves) thoracic and thoracolumbar/lumbar curves, with a curve magnitude difference less than 10 degrees°. The concave and convex sides of the paraspinal muscle region were identified based on ultrasound images obtained from the Scolioscan. The demographic data of the subjects are shown in Table 1.

**Figure 2:**
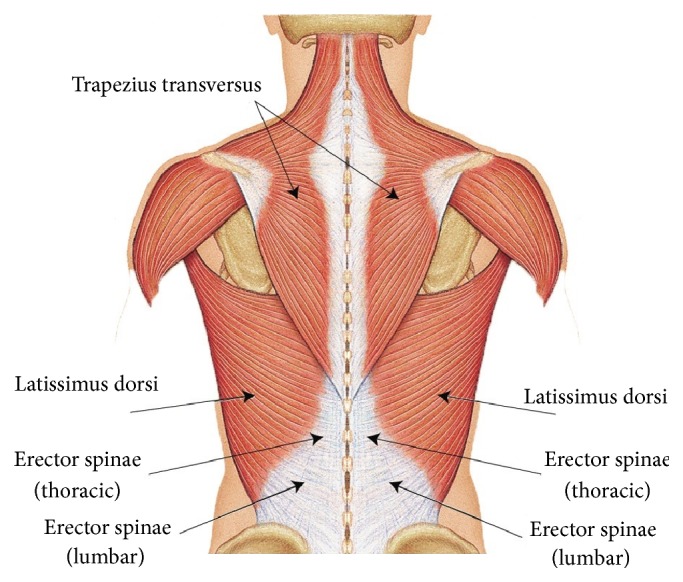
Placement of the EMG electrodes at the targeted paraspinal muscle regions.

The parameters of the sEMG assessment were formulated based on the Surface EMG for Noninvasive Assessment of Muscles (SENIAM) standards [25]. The sEMG activity was acquired with the use of a preamplified sensor, MyoScan (model T9503M), and a data acquisition system, Flexcomp (model T7555M), both from Thought Technology (Montreal, Canada). The sEMG electrodes with ground reference from the same company (Triode T3402M) were placed onto the paraspinal muscles, namely, the trapezius, latissimus dorsi, erector spinae at the thoracic region, and erector spinae at the lumbar region (Figure 2) in pairs to determine the muscle activity along the whole spine. The back of the subject was shaved and cleaned with alcohol as suggested by the SENIAM standards. The electrodes were placed by a physiotherapist based on the SENIAM instructions and the sEMG signals were verified by an impedance test found in the BioGraph Infiniti software (Thought Technology Ltd.). The EMG assessment was only performed when the impedance check indicated that the data received were under 50 khms/s.

**Figure 3:**
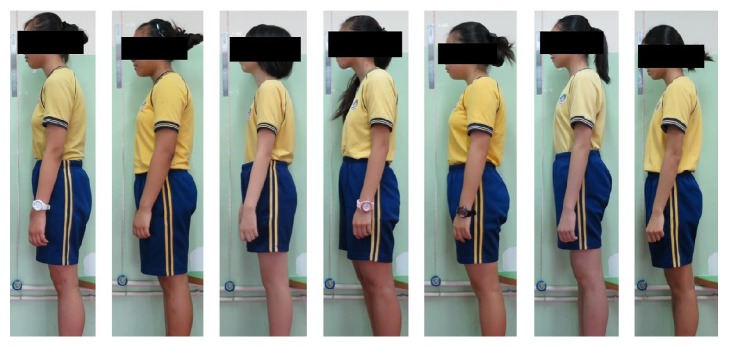
Habitual standing postures of participants.

Figure 3 shows the habitual standing postures of some of the selected participants. It can be observed that the common posture problems are kyphosis, flat back, rounded and elevated shoulders, and a pushed-forward head position.

During the collection of the sEMG data for habitual standing, the study participants were barefoot with arms relaxed and lightly clasped in front of their body and feet positioned 20 cm apart. They were instructed to focus straight ahead and look at a designated point [26]. The habitual postures are the natural postures of the subjects performed without any instructions from the physiotherapist. An adjustable height treatment table was used for all the habitual sitting positions. The hips and knees were flexed to 90°. Under standardized instructions, the participants were positioned by the same investigator for all of the trials.

**Figure 4:**
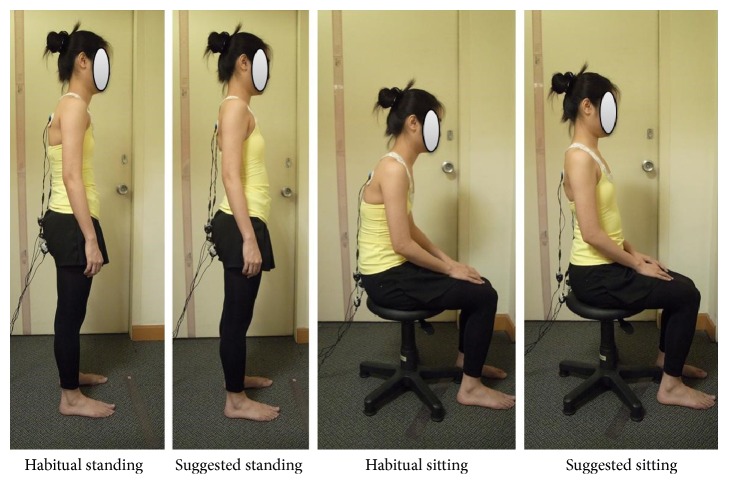
Subject whose posture has been “corrected” per instructions from a physiotherapist.

The requirements of the standing and sitting positions were used per recommendations in McKenzie [27] and Cheung et al. [28], and the subjects were guided to perform the posture accordingly by the physiotherapist (as shown in Figure 4).

1. Suggested standing position: the head and ankles should be straight, shoulders and hip are level, kneecaps face the front, the head and knees are straight, and the chin should be parallel to the floor and aligned with the ears. The lower back should be slightly bent forward with the aid of the chest, stomach, and buttock muscles.
2. Suggested sitting position: the head and ankles should be straight, shoulders and hips are level, kneecaps face the front, and the chin should be parallel to the floor and aligned with the ears. The lower back should be slightly bent forward to support the body with no extra weight distributed onto the spine.

The measurements of the EMG activity of the paraspinal muscles of the subjects were taken during the habitual postures and suggested positions of standing and sitting for a duration of 1 minute and repeated twice. A band pass filter that ranged from 10 to 500 Hz was applied to eliminate undesired artifacts, such as sudden movement, and a 60 Hz notch filter was used to eliminate noise. The sEMG signals were sampled at a rate of 2048 Hz. The EMG raw data were averaged by using root mean square (RMS) to obtain the average amplitude of the EMG signal. The RMS sEMG ratio of the subjects was calculated based on the following equation [29]:

(1) $$\begin{array}{c} \text{RMS sEMG Ratio}=\frac{\text{RMS sEMG}\left(\text{convex}\right)}{\text{RMS sEMG}\left(\text{concave}\right)}. \end{array}$$

The ratio is an index of the symmetric sEMG activity of the tested muscles, in which when the ratio is 1, the tested pairs of muscles have identical sEMG activity from the concave and convex sides of the tested muscle. If the ratio is less than 1, the concave side of the muscle has stronger sEMG activity than the convex side. If the ratio is larger than 1, the concave side of the muscle has weaker sEMG activity than the convex side. The equation was applied to assess the effectiveness of the suggested positions for the scoliosis subjects. The suggested positions are effective if the ratio is closer to 1 compared to the ratio recorded for the habitual postures.

## 3. Statistics

Statistical analyses were conducted by using the SPSS 19 program for Windows. The difference between the convex and concave sides during habitual standing and sitting was compared by *t*-testing with the significance level at *p* < 0.05. Besides, the difference between habitual and the suggested standing and sitting sEMG ratios was compared through the significance of a one-sample *t*-test with a test value of 1. The level of significance is *p* < 0.05.

## 4. Results

Table 2 shows the results of the mean RMS sEMG values (S.D.) of the paraspinal muscles of the subjects during habitual standing and sitting. The data are categorized based on PUMC type and occurrence of spinal curvature on the convex side of the subjects.

Table 3 shows the mean of the RMS sEMG ratio values (±*s*) of the paraspinal muscles of the subjects during habitual standing and sitting and the suggested standing and sitting positions. The highlighted parts indicate posture improvement under the guidance of the physiotherapist, in which the RMS sEMG ratio of the suggested positions is closer to 1 as opposed to that obtained by the same habitual posture.

## 5. Discussion

Based on the acquired data in Table 2, it can be observed that, for the habitual postures, the convex side of the paraspinal muscles tends to have stronger RMS sEMG values than the concave side at certain regions where spinal curvature has occurred (in bold and italic in Table 2). This situation is found to be true for both the PUMC type Ia and IIc subjects. This indicates that the curvature of the spine has affected the paraspinal muscle activity and caused muscle impairment. For example, for the PUMC type IIc subjects, the spinal curve is found at the thoracic and lumbar regions, with the convex to the right side at the thoracic region and to the left side at the lumbar region. The average RMS sEMG values at the right side of the trapezius and latissimus dorsi are stronger than those at the left side, and the average RMS sEMG values at the left side of the erector spinae thoracic are stronger than those at the right side. For the PUMC type Ia subjects, the convex side of their spinal curvature is on the right side, and the average RMS EMG values on the right side for all tested muscle regions are stronger than those on the left side. In terms of the latissimus dorsi region during habitual standing, the convex and concave sides have a significant difference (*p* = 0.043). This is consistent with the findings of Mannion et al. where the concave side of the paraspinal muscles has lower bioelectric activity which caused muscle impairment at the convex side [30].

However, a contradictory situation is found in the PUMC type Ib subjects for most of the tested regions; the RMS EMG values show a different result than that for the PUMC type Ia and IIc subjects. The RMS EMG values at the muscle region on the concave side are larger than those on the convex side. This may be due to the comparatively smaller degree of spinal curvature of the PUMC type Ib subjects (mean = 13.6); therefore, the reduction of bioelectric activity on the concave side was not reflected from the sEMG data. It is important to note that the convex side where the spinal deformity has taken place does not necessarily incur stronger sEMG values as opposed to the concave side. An influencing factor to take into consideration could be due to the degree of the spinal curvature.

The treatment for adolescents with mild idiopathic scoliosis is often passive, with only periodical observation by orthopaedists. It is possible that scoliotic adolescents suffer from progressive spinal curvature in a short period of time during puberty [31]. Adolescents with an idiopathic scoliosis curve that is over 21° are usually recommended to wear a brace made of rigid materials, such as that with a plastic or metal frame, to limit the progression of the spinal deformity. The brace causes irritation to the wearer, thus resulting in deterioration in the quality of life [32]. In some of the more severe cases, surgery may be needed to rebuild the spine by fusing bone grafts to the spine discs. This type of surgery has significant impacts on patients, which results in their immobility and therefore inability to carry out daily activities.

Despite the suggested therapy in which patients with mild idiopathic scoliosis should be periodically observed rather than prescribed with any type of treatment or exercise during the early stages, unless a rapid change takes place in the curvature angle or there is spine rotation, adolescents with mild scoliosis could be treated with the use of exercise, as indicated by work in the literature; see [33–35]. In 1984, Dickson [36] provided a critical review on the use of exercise for the treatment of scoliosis. In the article, examples of successful cases in which scoliosis was treated with exercise were provided [37], and by adding loads to recover the postural balance of the patients, “the spinal deformity can be completely eliminated” [36]. As mentioned, Chwała et al. [16] performed an EMG assessment of adolescents with idiopathic scoliosis, and the results suggested that patients with a single curve show a beneficial corrective factor during asymmetric load-free and symmetric exercises.

In this study, the subjects are asked to perform habitual standing and sitting and given suggested standing and sitting positions, which are recorded with the EMG method. The suggested positions as guided by a physiotherapist with the aim of retaining postural balance of the subjects during standing and sitting are considered to be common daily positions. By restricting the scoliotic adolescents to a balanced posture, the paraspinal muscles between the two sides of the spine were able to achieve a more balanced state.

The results in Table 3 show that the PUMC type Ib subjects, with a single thoracolumbar curve, benefit relatively more from the suggested positions. During the suggested sitting position, they are able to achieve a more balanced RMS EMG ratio (closer to 1) at the trapezius, erector spinae thoracic, and erector spinae lumbar regions versus in their habitual sitting posture. During the suggested sitting position, for PUMC types Ia and Ib subjects, the RMS EMG ratio is closer to 1 at the trapezius, erector spinae thoracic, and erector spinae lumbar regions versus when in the habitual sitting posture. The result suggests a similar finding as that in Chwała et al. [16] in that the single curve patients with idiopathic scoliosis benefit most during static exercise compared to those with double curve scoliosis.

Overall, during habitual standing, the RMS sEMG ratio for the trapezius region has a significant difference with 1 (*p* = 0.023) while none of the tested muscle regions have a ratio with a significant difference with 1 during the suggested standing posture. During habitual sitting, the ratio for both the trapezius area and erector spinae at the thoracic region tends to have a significant difference of 1 (*p* = 0.053 and *p* = 0.076, resp.) while during the suggested sitting posture, only the ratio of the trapezius has a significant difference with 1 (*p* = 0.025). The results show that the subjects can achieve a more balanced sEMG signal during the suggested posture while standing and sitting.

Based on the presented findings, it is considered that, through the motor learning ability of those with AIS, postural balance can be permanently maintained by practicing the suggested positions on a regular basis as a new motor task [38].

## 6. Conclusions

The aim of this study is to conduct a thorough evaluation of the myoelectric activity of those with AIS during habitual standing and sitting and comprises part of our research work for the formulation of a database towards sEMG biofeedback training. The major findings of this study are as follows.

1. The results from the PUMC type Ia and IIc subjects reflect consistency with stronger RMS sEMG values from the convex side of the paraspinal muscles as opposed to the concave side.
2. The correction of posture per instructions from a physiotherapist can reduce muscle impairment as evidenced by the RMS sEMG ratios, and the result indicates that the PUMC type Ia and Ib subjects with a single thoracic or thoracolumbar curve benefit relatively more from the suggested positions. The result also echoes a similar finding in the literature on treating scoliosis patients with static exercises. The single curve patients benefit more and the paraspinal muscles between the two sides of their spine are better balanced than their counterparts with a double curve during symmetrical exercise.

The findings can be used to provide a less intrusive type of early intervention and serve as a means of care for adolescents with mild idiopathic scoliosis (i.e., Cobb's angle less than 20 degrees) and therefore reduce the possible need to prescribe brace wear treatment due to its associated psychological stress and negative impacts on adolescents. It is considered that, through motor learning of the suggested positions as a new type of motor task, the concave and convex sides of the paraspinal muscles will be more balanced.

## Figures and Tables

**Table 1:** Demographic data of subjects with ATI ≥ 3°.

PUMC type	Convex side	*N* =		Mean (S.D.)
Ia	Right (thoracic)	4	Height (cm)	156.5 (5.00)
			Weight (kg)	47.8 (5.34)
			Thoracic curve angle (°)	17.6 (8.02)
			Lumbar curve angle (°)	0

Ib	Left (thoracolumbar)	2	Height (cm)	153.5 (3.54)
			Weight (kg)	45.4 (11.7)
			Thoracic curve angle (°)	13.6 (0.85)
			Lumbar curve angle (°)	0

IIc	Right (thoracic)		Height (cm)	150.1 (5.98)
		7	Weight (kg)	42.89 (7.72)
	Left (lumbar)		Thoracic curve angle (°)	15.8 (5.21)
			Lumbar curve angle (°)	16.7 (4.03)

**Table 2:** Result of mean RMS sEMG values (S.D.) of the paraspinal muscles of subjects during habitual standing and sitting.

PUMC type	Convex side	*N* =	Muscle region	Mean RMS sEMG (S.D.) (*μ*V)			
				Habitual standing (left)	Habitual standing (right)	Habitual sitting (left)	Habitual sitting (right)
Ia	Right (thoracic)	4	Trapezius	3.00 (2.21)	**4.90** (**2.30**)	2.65 (1.23)	**5.73 (4.24)**
			Latissimus dorsi	2.75 (1.70)	**4.22** (**1.12**)	3.13 (1.55)	**5.16 (1.79)**
			Erector spinae thoracic	2.68 (0.62)	**6.06** (**6.88**)	3.95 (1.71)	**11.5 (13.4)**
			Erector spinae lumbar	3.24 (3.17)	**5.30** (**5.91**)	4.95 (5.11)	**6.02 (4.56)**

Ib	Left (thoracolumbar)	2	Trapezius	2.62 (0.81)	*7.93* (*1.29*)	2.54 (1.39)	*6.37* (*2.33*)
			Latissimus dorsi	6.15 (2.41)	*7.30* (*1.62*)	6.95 (4.14)	*7.99 (3.02) *
			Erector spinae thoracic	4.94 (2.44)	*5.50* (*1.91*)	10.3 (1.04)	5.82 (1.93)
			Erector spinae lumbar	4.06 (1.57)	*19.5* (*13.1*)	5.77 (2.04)	*11.9* (*10.4*)

IIc	Right (thoracic)		Trapezius	1.71 (1.04)	**4.94** (**6.40**)	2.40 (1.55)	**5.06** (**5.13**)
		7	Latissimus dorsi	3.57 (2.19)	**4.58** (**2.72**)	4.03 (2.30)	**4.44** (**1.90**)
	Left (lumbar)		Erector spinae thoracic	**3.06** (**1.99**)	2.83 (1.17)	**9.39** (**7.25**)	8.02 (4.67)
			Erector spinae lumbar	**3.68** (**2.38**)	3.31 (1.75)	4.71 (2.72)	5.15 (3.19)

**Table 3:** Mean of RMS sEMG ratio values (±*s*) of the paraspinal muscles of subjects during habitual standing and sitting.

PUMC type	Concave side	*N* =	Muscle region	Mean RMS EMG ratio (±*s*)			
				Habitual standing	Suggested standing	Habitual sitting	Suggested sitting
Ia	Right (thoracic)	4	Trapezius	2.54 ± 1.95	4.22 ± 5.87	3.84 ± 3.95	**3.16 ± 2.16**
			Latissimus dorsi	2.00 ± 1.22	2.41 ± 1.64	2.22 ± 1.66	2.32 ± 2.06
			Erector spinae thoracic	1.87 ± 1.70	2.00 ± 1.49	2.83 ± 2.69	**2.51 ± 2.68**
			Erector spinae lumbar	1.70 ± 0.85	3.05 ± 2.45	3.50 ± 4.88	**2.66 ± 2.92**

Ib	Left (thoracolumbar)	2	Trapezius	0.34 ± 0.16	**0.48 ± 0.47**	0.38 ± 0.08	**0.53 ± 0.19**
			Latissimus dorsi	0.90 ± 0.53	0.60 ± 0.15	0.83 ± 0.20	0.75 ± 0.28
			Erector spinae thoracic	0.87 ± 0.14	**0.99 ± 0.67**	1.84 ± 0.43	**1.34 ± 0.74**
			Erector spinae lumbar	0.30 ± 0.29	**0.91 ± 0.43**	0.91 ± 0.97	**1.03 ± 0.97**

IIc	Right (thoracic)		Trapezius	2.73 ± 1.76	3.76 ± 4.87	2.39 ± 1.63	1.96 ± 0.94
		7	Latissimus dorsi	2.46 ± 2.90	**1.79 ± 1.76**	1.97 ± 2.63	2.50 ± 3.88
	Left (lumbar)		Erector spinae thoracic	1.36 ± 1.14	**1.10 ± 0.67**	1.48 ± 1.35	**1.35 ± 1.26**
			Erector spinae lumbar	1.21 ± 0.59	1.01 ± 0.65	1.21 ± 1.25	1.22 ± 1.55
